# The effect of fixation type on the survivorship of contemporary total knee arthroplasty in patients younger than 65 years of age: a register-based study of 115,177 knees in the Nordic Arthroplasty Register Association (NARA) 2000–2016

**DOI:** 10.1080/17453674.2019.1710373

**Published:** 2020-01-13

**Authors:** Mika J Niemeläinen, Keijo T Mäkelä, Otto Robertsson, Annette W-Dahl, Ove Furnes, Anne M Fenstad, Alma B Pedersen, Henrik M Schrøder, Aleksi Reito, Antti Eskelinen

**Affiliations:** a Coxa Hospital for Joint Replacement, and Faculty of Medicine and Health Technologies, University of Tampere, Tampere, Finland;; b Finnish Arthroplasty Register, National Institute for Health and Welfare, Helsinki, Finland;; c Department of Orthopaedics and Traumatology, Turku University Hospital, Turku, Finland;; d The Swedish Knee Arthroplasty Register, Department of Orthopedics, Skane University Hospital, Lund, Sweden;;; e The Norwegian Arthroplasty Register, Department of Orthopaedic Surgery, Haukeland University Hospital, Bergen, Norway;; f Department of Clinical Medicine, University of Bergen, Haukeland University Hospital, Bergen, Norway;; g Department of Clinical Epidemiology, Aarhus University Hospital. Denmark and Danish Knee Arthroplasty Registry;; h Department of Orthopaedic Surgery, Naestved Hospital, Denmark;; i Department of Clinical Sciences, Orthopedics, Lund University, Sweden

## Abstract

Background and purpose — Cemented fixation is regarded as the gold standard in total knee arthroplasty (TKA). Among working-age patients, there has been controversy regarding the optimal fixation method in TKA. To address this issue, we conducted a register-based study to assess the survivorship of cemented, uncemented, hybrid, and inverse hybrid TKAs in patients aged < 65 years.

Patients and methods — We used the Nordic Arthroplasty Register Association data of 115,177 unconstrained TKAs performed for patients aged < 65 years with primary knee osteoarthritis over 2000–2016. Kaplan–Meier (KM) survival analysis with 95% confidence intervals (CI) and Cox multiple-regression model with adjustment for age, sex, and nation were used to compare fixation methods in relation to revision for any reason.

Results — The 10-year KM survivorship of cemented TKAs was 93.6% (95% CI 93.4–93.8), uncemented 91.2% (CI 90.1–92.2), hybrid 93.0% (Cl 92.2–93.8), and inverse hybrid 96.0% (CI 94.1–98.1). In the Cox model, hybrid TKA showed decreased risk of revision after 6 years’ follow-up compared with the reference group (cemented) (hazard ratio [HR] 0.5 [CI 0.4–0.8]), while uncemented TKAs showed increased risk of revision both < 1 year (HR 1.4 [1.1–1.7]) and > 6 years’ (HR 1.3 [1.0–1.7]) follow-up compared to the reference.

Interpretation — Both cemented and hybrid TKAs had 10-year survival rates exceeding 92–>93% in patients aged < 65 years. Cemented TKA, however, was used in the vast majority (89%) of the operations in the current study. As it performs reliably in the hands of many, it still deserves the status of gold standard for TKA in working-age patients.

Previous studies reported both highest increase in incidence of TKAs and also highest risk for revision in patients younger than 65 years of age (Julin et al. 2010, Carr et al. 2012, Leskinen et al. 2012, Meehan et al. 2014, Nemes et al. 2015, Niemelainen et al. 2017). This has increased the interest in finding a more durable fixation method for TKA. A previous systematic review did not report any differences in survival or functional outcome between cemented and uncemented TKAs in patients aged 60 years or less (Franceschetti et al. 2017). A meta-analysis without age limit showed better survival rates with cemented TKAs when all studies were combined, but in randomized studies survivals were equivocal (Gandhi et al. 2009). Uncemented fixation in TKA has offered outcomes comparable with cemented TKA in a few studies, but higher costs of uncemented components have favored cemented TKA still as gold standard (Dalury 2016, Miller et al. 2018, Zhou et al. 2018).

A previous study applying radiostereometric analysis (RSA) showed that early migration seen with uncemented tibial components settled until 2 years whereas cemented ones continued to migrate (Wilson et al. 2012, Henricson and Nilsson 2016). So far, the use of uncemented TKAs has been limited. Previous studies have reported an increased risk for aseptic loosening of the tibial component in patients treated with uncemented TKA (Bassett 1998, Duffy et al. 1998, Berger et al. 2001a, Goldberg and Kraay 2004, Carlsson et al. 2005), but due to evolvement of designs and materials uncemented fixation has become an interesting choice, especially for younger patients with good bone quality (Hu et al. 2017). Trabecular metal (TM) has showed promising results in both register and clinical studies (Niemelainen et al. 2014, Henricson et al. 2013, Pulido et al. 2015). Although differences have been observed between different fixation concepts in terms of revision rates, functional outcomes have been equivalent irrespective of the fixation method (Gandhi et al. 2009, Gao et al. 2009, Demey et al. 2011, Arnold et al. 2013). The optimal fixation method in TKA still remains controversial for these younger patients.

We assessed survivorships of 4 different fixation methods (cemented, uncemented, hybrid, and inverse hybrid) in patients younger than 65 years of age based on the Nordic Arthroplasty Register Association (NARA) database.

## Patients and methods

**Figure 1.**
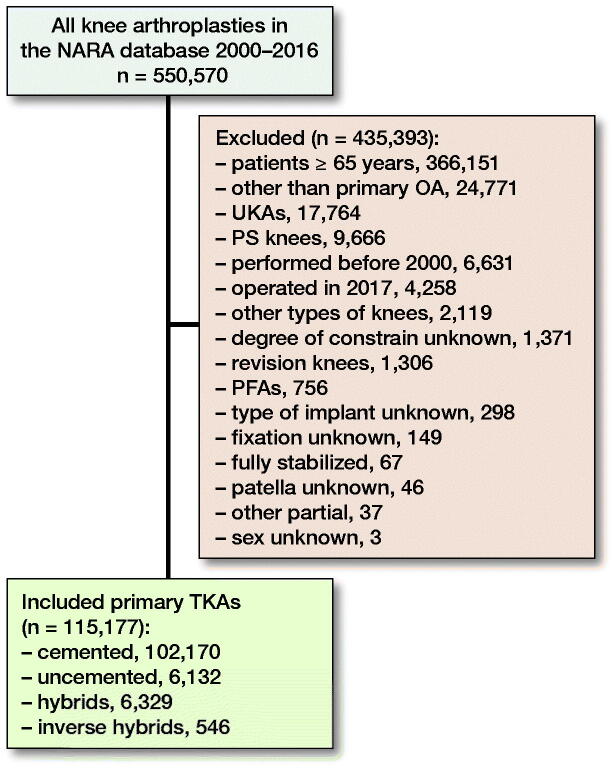
Flow chart.

We included all uni- or bilateral unconstrained primary TKAs that had been implanted in patients aged less than 65 years for primary OA over 2000–2016 (Figure 1). Previous reports have shown that the effect of including bilateral cases in studies of hip and knee joint prosthesis survival is negligible (Robertsson and Ranstam 2003, Lie et al. 2004). The Swedish Knee Arthroplasty Register (SKAR), the Danish Knee Arthroplasty Register (DKR), the Norwegian Arthroplasty Register (NAR), and the Finnish Arthroplasty Register (FAR) participated in the study. The Nordic Arthroplasty Register Association (NARA) compiles data on 4 Nordic countries that have similar healthcare organizations and comparable patient characteristics (Robertsson et al. 2010). A NARA minimal dataset was created to contain data that all 4 registers could deliver (NARA report 2016). The NARA dataset includes 20 different main variables and in total 90 variables. All registers use individual-based registration of operations. Selection and transformation of the respective data sets and de-identification of the patients, which included the deletion of personal identity numbers, were performed within each national register. The anonymous data were then merged into a common database. Data were treated with full confidentiality, according to the rules of the respective countries. The quality of data in the Nordic registers is high, including both 100% coverage and the following completeness: SKAR 97%, DKR 97%, NAR 97%, FAR 96% (NARA report 2016) (Espehaug et al. 2006). The fixation of TKAs was divided into 4 groups: (1) cemented, (2) uncemented, (3) hybrid (uncemented femur with cemented tibia), and (4) inverse hybrid (cemented femur with uncemented tibia).

### Statistics

We assessed the descriptive statistics of the patients included. The inclusion time period was 2000–2016. We used Kaplan–Meier (KM) survival analysis to assess implant survival probability (with respective 95% confidence interval [CI]) of the TKA fixation at 10 and 15 years. The results in tables and figures were not shown when less than 40 knees were at risk. Outcome was defined as removal, addition, or exchange of at least one of the components, including polyethylene insert exchanges of modular tibial components, for any reason.

We used Cox regression analysis to estimate hazard ratios associated with implant survival. Covariates included in the analysis were fixation type, sex, country, and age. Age was included as a continuous variable whereas the others were categorical. Correlation of scaled Schoenfeld residuals with time was examined to investigate violation of proportional hazard (PH) assumption. Log–log survival curves were also inspected visually to see if assumption was met. We detected multiple violations of PH assumption. In order to deal with PH violation, we used time-dependent coefficients (fixation, age, sex, and nation) using step function. Based on the log–log curves cut-offs were set as follows: 1, 3, and 6 years. We did stratified analyses based on age and implant brand group and similar time axis division was made according to log–log curves and residual testing. For the time dependent coefficients the data were broken into time-dependent parts according to the time intervals used in the time axis division. For each final analysis the PH test investigating Schoenfeld residuals was performed.

Statistical analyses were performed using R 3.5.2, survival package (R Foundation for Statistical Computing, Vienna, Austria).

### Ethics, data sharing, funding, and potential conflicts of interest

Ethical approval for the study was obtained through the ethical approval process of each national registry: the Ethics Board of Lund University (LU20-02) (Sweden), the National Institute of Health and Welfare (Dnro THL/1743/5.05.00/2014) (Finland), the Norwegian Data Inspectorate (ref 24.1.2017: 16/01622-3/CDG) (Norway) and the Danish Data protection agency (1-16-02-54-17) (Denmark).

No funding was received. Authors did not have any conflicts of interest. Data sharing is not possible.

## Results

**Figure 2.**
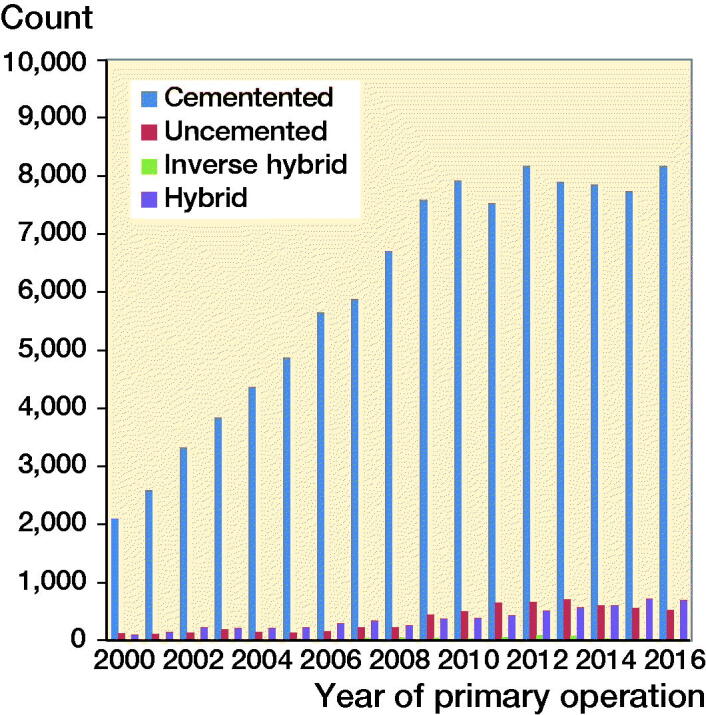
Number of operations.

The mean follow-up time standard deviation (SD) was 6.4 (4.3) years for cemented TKA, 4.7 (3.4) years for uncemented TKA, 6.0 (4.3) years for hybrid TKA, and 6.1 (3.2) years for inverse hybrid TKA. There were slight differences in the proportion of men between the fixation groups, ranging from 40% in the cemented to 44% in the uncemented group (Table 1). TKA models varied between countries without a common trend and the most commonly used TKA models in the participating countries are given in Table 2 (see Supplementary data). Nexgen, PFC, and Triathlon were the most commonly used models within the fixation concepts (Table 3, see Supplementary data). The number of TKAs performed annually grew substantially over 2000–2009, and remained rather stable after that; cemented fixation was used in the vast majority of TKAs over the whole study period (Figure 2). Altogether, cemented fixation was used in 89% of all TKAs, and uncemented in 5.3%, hybrid in 5.5%, and inverse hybrid in 0.5%, respectively. The patella was resurfaced in 24,487 TKAs (21%) and uncemented patellar buttons were used in only 151 (0.1%) TKAs. In the subgroup of Nexgen TKAs, the patella was resurfaced in 5,821 (22%) TKAs, and an uncemented patellar button was used only in 2 knees (both of them in the cemented Nexgen group).

**Table 1.** Demographic data

		Fixation concept		
Uncemented	Inverse hybrid	Hybrid	Cemented	
No of TKAs (%)	6,132 (5.3)	546 (0.5)	6,329 (5.5)	102,170 (88.7)
Mean age, years (SD)	57 (5.6)	57 (5.4)	58 (5.2)	59 (4.9)
Men, %	44	42	41	40
Country, n of TKAs (%)				
Finland	900 (2.5)	350 (1.0)	146 (0.4)	34,406 (96)
Norway	1,191 (8.7)	10 (0.1)	1,981 (14)	10,565 (77)
Sweden	2,284 (5.0)	128 (0.3)	74 (0.2)	43,268 (95)
Denmark	1,757 (8.8)	58 (0.3)	4,128 (21)	13,931 (70)

**Figure 3.**
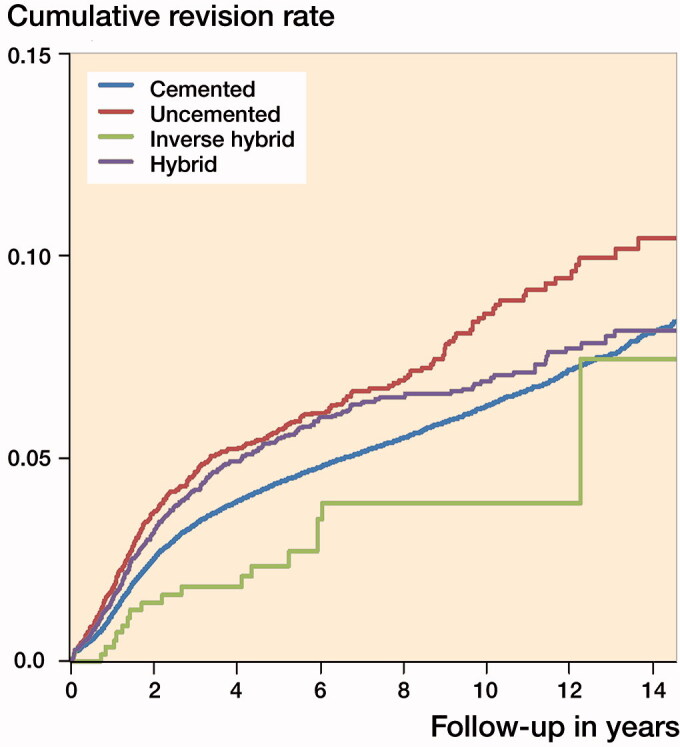
Unadjusted Kaplan–Meier cumulative risk of revision by fixation type in patients < 65 years of age.

At 15 years, KM-based survival rates were: cemented 91.3% (Cl 91.0–91.7), hybrid 91.4% (CI 90.2–92.6), uncemented 88.7% (CI 87.0–90.4). For inverse hybrid only 10-year survival was available (96.0% [CI 94.1–98.1]) (Table 4, Figure 3).

**Table 4.** Unadjusted Kaplan–Meier (KM) 10- and 15-year survival rates (%) with 95% confidence intervals (CI) for uncemented, inverse hybrid, hybrid, and cemented TKA

Type of fixation	No. of		10-year	K–M survivorship	15-year n at risk	K–M survivorship rate (CI)
	knees	revisions	n at risk	rate (CI)		
Uncemented	6,132	363	915	91.2 (90.1–92.2)	214	88.7 (87.0–90.4)
Inverse hybrid	546	16	66	96.0 (94.1–98.1)	–	–
Hybrid	6,329	330	1,349	93.0 (92.2–93.8)	239	91.4 (90.2–92.6)
Cemented	102,170	5, 040	24,954	93.6 (93.4–93.8)	4,259	91.3 (91.0–91.7)

In the Cox regression analysis, uncemented fixation showed an increased risk of revision compared with the reference group (cemented TKA) both during the first postoperative year and also after 6 years of follow-up. Hybrid fixation was associated with a decreased risk of revision compared with the cemented fixation after 6 years of follow-up. The risk of revision was similar between the inverse hybrid and the reference group (Table 5). Because of the age dependence of TKA survivorship, the additional Cox regression analyses were conducted for 2 different age groups: 55–64 years of age (Table 6) and less than 55 years of age (Table 7). In patients aged 55–64 years, risk of revision with uncemented TKAs was increased in comparison with the cemented reference group during the first 3 years of follow-up and after that similar compared with reference. Hybrid TKAs still showed a decreased risk of revision after 6 years of follow-up, a finding that was already seen in the whole study cohort (Table 5). In patients aged less than 55 years, revision risks were similar between fixation methods (Table 7). Differences between age, sex, and country were the other covariates in the Cox regression analysis and their results are listed in Table 8 (see Supplementary data).

**Table 5.** Cox regression with time-dependent coefficients (all patients aged < 65 years included, cemented TKA as reference)

Type of fixation	Follow-up (years)	Hazard ratio (95% CI)
Uncemented	< 1	1.38 (1.13–1.70)
	1–3	1.14 (0.97–1.35)
	3–6	0.95 (0.72–1.25)
	> 6	1.32 (1.00–1.73)
Inverse hybrid	< 1	0.29 (0.07–1.16)
	1–3	0.67 (0.34–1.35)
	3–6	0.91 (0.38–2.19)
	> 6	0.54 (0.13–2.15)
Hybrid	< 1	1.11 (0.88–1.39)
	1–3	0.94 (0.78–1.12)
	3–6	1.07 (0.82–1.40)
	> 6	0.54 (0.38–0.78)
Cemented		1.0 Reference

**Table 6.** Cox regression with time-dependent coefficients in patients aged 55–65 years

Type of fixation	Follow-up (years)	Hazard ratio (95% CI)
Uncemented	< 1.5	1.37 (1.13–1.67)
	1.5–3	1.31 (1.01–1.69)
	3–6	0.86 (0.59–1.24)
	> 6	1.32 (0.96–1.83)
Inverse hybrid	< 1.5	0.44 (0.14–1.37)
	1.5–3	0.65 (0.21–2.02)
	3–6	0.88 (0.28–2.75)
	> 6	0.49 (0.07–3.48)
Hybrid	< 1.5	1.15 (0.94–1.41)
	1.5–3	0.90 (0.68–1.20)
	3–6	1.14 (0.85–1.53)
	> 6	0.55 (0.37–0.83)
Cemented		1.0 Reference

**Table 7.** Cox regression with time-dependent coefficients in patients aged < 55 years

Type of fixation	Hazard ratio (95% CI)
Uncemented	1.10 (0.91–1.32
Inverse hybrid	0.62 (0.29–1.29
Hybrid	0.83 (0.67–1.04)
Cemented	1.0 Reference

The inverse hybrid group mainly comprised Nexgen TKAs (95% of the knees) (Table 3, see Supplementary data), and approximately more than 80% of the inverse hybrid Nexgen TKAs used TM monoblock tibial components (an estimate from national registers’ data). Because of the obvious risk for selection bias, we conducted an additional sensitivity analysis to diminish bias between groups. For this analysis, we included only patients operated on with Nexgen TKAs (Table 9, see Supplementary data). In this sensitivity analysis, survival rates of different fixations were in descending order: the inverse hybrid 96.6% (CI 94.7–98.5), cemented 95.8% (CI 95.5–96.1), uncemented 93.2% (CI 91.9–94.6), and hybrid 92.0% (CI 90.4–93.7) at 7 years (Table 10). In the Cox analysis of the Nexgen subgroup, increased risk of revision was found for uncemented and hybrid TKAs compared with cemented TKAs, and for inverse hybrid TKAs the risk of revision was comparable to cemented TKAs (Table 11).

**Table 10.** Unadjusted Kaplan–Meier 7- and 10-year survival rates with 95% confidence intervals for uncemented, inverse hybrid, hybrid, and cemented TKA in the Nexgen subgroup

Type of fixation	No. of		7-year n at risk	K–M survivorship rate (CI)	10-year n at risk	K–M survivorship rate (CI)
	knees	revisions				
Uncemented	2,311	114	238	93.2 (91.9–94.6)	–	–
Inverse hybrid	497	13	185	96.6 (94.7–98.5)	55	96.6 (94.7–98.5)
Hybrid	1,629	91	155	92.0 (90.4–93.7)	–	–
Cemented	27,934	901	8,477	95.8 (95.5–96.1)	3,691	94.9 (94.6–95.3)

**Table 11.** Cox regression with time-dependent coefficients in patients aged < 65 years in the Nexgen subgroup

Type of fixation	Hazard ratio (95% CI)
Uncemented	1.37 (1.12–1.67)
Inverse hybrid	0.59 (0.34–1.03)
Hybrid	1.47 (1.16–1.87)
Cemented	1.0 Reference

## Discussion

We found that both cemented and hybrid TKAs showed 10-year survival rates exceeding 92–>93% in patients aged < 65 years. Even though hybrid/inverse hybrid versions of the well-performing contemporary TKA designs provided younger patients with a good mid-term outcome in our study, they were still used in a limited number of patients. And especially in the inverse hybrid group, one single TKA design with a very good track record comprised the vast majority of the whole group. It is thus safe to conclude that cemented TKA still fulfils the most important task of a TKA: it works very reliably in the hands of many. Also, cemented TKA should still be considered as the gold standard in TKA of all OA patients irrespective of their age.

We acknowledge certain strengths and limitations in our study. The major strength of our study is the unique collaboration of 4 national registers in the creation of a multinational database comprising a high number of patients. This NARA database enables international comparisons to reveal possible differences in trends and outcomes of TKA. To our knowledge, this is the first multi-national, register-based study comparing the outcomes of all 4 fixation methods in TKA. There are also a few obvious limitations in our study. First, there were clearly fewer patients in the alternative fixation groups as compared with the cemented reference group (Figure 2). There are potential sources of selection bias in our data. Other concepts than cemented TKAs may have been done in higher volume units, and there may have been less preoperative bone loss or less severe deformity. On the other hand, uncemented components may have been used in patients with higher demands and also there may have been concerns about cemented fixation during operation. If the choice of fixation had been constant at hospital level in our study population, this might lower this risk of bias. Further, especially inverse hybrid fixation, but also hybrid fixation to some extent, had another obvious advantage over cemented fixation in our study setting. That is the monoblock uncemented tibial component, since wash-out procedures for infection in such knees (without exchange of any component) have not been regarded as revisions in the NARA data. Thus, due to a small number of patients and also the possibility of some missing infection revisions, the results of inverse hybrids should be interpreted with caution. Further, Nexgen TKAs comprised 91% of the inverse hybrid group. This implant has been reported to have 97–99% 10-year survival rate in previous studies (Kim et al. 2012, Niemelainen et al. 2014, Robertsson et al. 2020). Further, in Finland Nexgen inverse hybrid TKAs (with TM tibial component) have been performed in only 3 hospitals, 1 of which is a high-volume specialized center (Niemelainen et al. 2014). In the hybrid group, 3 TKA designs with a very good track record (PFC, Nexgen, Profix) comprised 76% of all TKAs. The second limitation is that, due to the nature of the NARA dataset, we had a limited number of covariates for analysis and also we did not have exact information on whether some of the uncemented implants were hydroxide apatite coated or not. On the hip side, HA coating does not seem to provide any added value in terms of improved survival rates (Hailer et al. 2015, Lazarinis et al. 2017), thus it most probably does not cause any bias to these TKA results.

In our study, the vast majority of TKAs performed for younger patients in the 4 Nordic reporting countries were still cemented, and very small changes, if any, were observed in the fixation methods used over the study period (Figure 2). The same trend in general has also been reported from other national registers: the annual report 2017 of the National Joint Registry for England, Wales and Northern Ireland (NJR) reported that the proportion of all cemented TKA implants increased from 82% in 2003 to 87% in 2016 (NJR annual report 2017). During the same time period uncemented implants decreased from 6.7% to 2.0% and hybrid implants from 2.8% to 0.4%. The same increasing trend of using cemented implants was seen in the Australian Joint Registry (AOANJRR annual report 2017). In our study, the proportion of cemented TKAs decreased only slightly from 96% in 2000 to 91% in 2016, and a simultaneous small increase in usage of uncemented TKAs was seen (from 2.5% to 6.5%, respectively).

In our study, both cemented and hybrid TKAs had up to 15-year survival rates exceeding 91% in patients aged < 65 years. Hybrid TKAs showed decreased risk of revision in comparison with cemented TKA after 6 years of follow-up. Inverse hybrid TKAs showed 96% survivorship at 10 years. Uncemented TKAs had the worst 10-year survival rate (91%). These findings are in line with previous literature. In a Finnish register-based study, uncemented TKAs had 1.4 times elevated adjusted hazard ratios (HR) for revision for any reason compared with cemented TKAs (Julin et al. 2010). In the AOANJRR annual report in 2017, the cumulative 10-year revision probability of minimally stabilized TKA was 4.5 (4.3–4.6) with cemented TKA, 6.1 (5.9–6.3) with uncemented TKA, and 4.6 (4.4–4.7) with hybrid TKAs. In the New Zealand Joint Register annual report in 2017, the revision rate with patient 55–64 years old was the highest with an uncemented implant: 0.84 (CI 0.67–1.05)/100 component-years compared with 0.62 (CI 0.58–0.66)/100 component-years with cemented implants and 0.61 (CI 0.47–0.77)/100 component-years with hybrid implants. To our knowledge, this study is the first to compare the survivorships of all 4 different fixation concepts in TKA.

In theory, younger patients might benefit from biologic fixation, i.e., bone ingrowth into uncemented implants. A meta-analysis (Gandhi et al. 2009) based on 5 RCTs and 10 observational studies, with different mean ages of patients and with a minimum follow-up of 2 years, found improved survival for cemented compared with uncemented implants when revision for aseptic loosening was used as an endpoint. Another systematic review and meta-analysis (Voigt and Mosier 2011) compared hydroxyapatite-coated, porous coated, and cemented tibial components. Evidence of more stable fixation after 2 years with hydroxyapatite-coated components compared with porous-coated and cemented implants was found, but revision rates at 10 year follow-up were similar. In an RCT no revision rates and survival were similar between the cemented and uncemented TKAs with mean follow-up of 15 years (Baker et al. 2007). In a systematic review of 11 RCTs to identify whether there was an association between fixation method and clinical outcome, it was found that short- and long-term outcomes were not influenced by fixation type (Arnold et al. 2013). In previous studies, early failures of uncemented TKAs were mainly caused by aseptic loosening of the patellar button and the tibial component (Collins et al. 1991, Bassett 1998, Duffy et al. 1998, Berger et al. 2001b, Barrack et al. 2004, Goldberg and Kraay 2004, Carlsson et al. 2005). Uncemented fixation has been associated with a high failure rate due to inadequate bone ingrowth in TKAs (Lombardi et al. 2007).

As stated earlier, Nexgen comprised 95% of the TKAs in the inverse hybrid group, and 87% of these Nexgen TKAs had been used with TM tibial components, which are known to have good results (Niemelainen et al. 2014). We tried to tackle the obvious possibility of selection bias by conducting a sensitivity analysis including only patients with Nexgen TKAs (Tables 10 and 11). In that analysis, it appeared that there was no statistically significant difference in mid-term survival rates or Cox-adjusted revision risks between inverse hybrid and cemented Nexgen TKAs. Further, hybrid and uncemented fixation showed an increased risk for revision in this Nexgen subgroup. Thus, the more expensive uncemented/hybrid/inverse hybrid versions did not provide these younger patients with any advantage over cemented fixation in the 10-year follow-up of Nexgen TKAs.

To conclude, cemented TKA still deserves the status of gold standard in TKA irrespective of the patients’ age. In addition to age, the optimal fixation method in younger patients may also be influenced by patients’ other characteristics such as level of activity, anatomy, or bone quality. Even though hybrid/inverse hybrid versions of the well-performing contemporary TKA designs provided younger patients with a good mid-term outcome in our study, these results do not support systematic use of these more expensive components in TKA for younger patients.

## Supplementary data

Tables 2, 3, 8, and 9 are available as supplementary data in the online version of this article, http://dx.doi.org/10.1080/ 17453674.2019.1710373

Study design: MN, AE. Analysis of data and statistics: AR, AE, MN. Review and interpretation of the results: MN, KM, OR, AW-D, OF, AF, AP, HS, AR, AE. Revision and approval of the final manuscript: MN, KM, OR, AW-D, OF, AF, AP, HS, AR, AE.

*Acta* thanks Alexander Liddle and Ola Rolfson for help with peer review of this study.

## Supplementary Material

Supplemental Material
