# Supplementary material for: The effect of fixation type on the survivorship of contemporary total knee arthroplasty in patients younger than 65 years of age: a register-based study of 115,177 knees in the Nordic Arthroplasty Register Association (NARA) 2000–2016
Source: Acta Orthop. 2020 Jan 13;91(2):184–90. doi: 10.1080/17453674.2019.1710373 (PMC7144225; doi:10.1080/17453674.2019.1710373)
Supplement: Supplemental Material [file IORT_A_1710373_SM6808.pdf]

Supplementary data

Table 2. The 5 most commonly used TKA designs in each country 2000–2016

Norway design	n	Sweden design	n	Finland design	n	Denmark design	n
LCS Complete	3,436	NexGen	16,734	Triathlon	12,834	PFC	6,967
Profix	3 319	PFC	12 984	Nexgen	7,515	Nexgen	4,407
Nexgen	3,034	AGC	3,635	PFC	5,168	AGC	3,426
LCS	816	Triathlon	3,393	Duracon	3,995	Vanguard	2,354
Triathlon	757	Vanguard	3,074	Vanguard	2,878	Advance	751

Table 3. The 5 most commonly used TKA designs in the fixation concepts over 2000–2016

Cemented design	n	Uncemented design	n	Hybrid design	n	Inverse hybrid design	n
Nexgen	27,948	Nexgen	2,204	PFC	2,248	Nexgen	497
PFC	22,360	Triathlon	1,375	Nexgen	1,629	PFC	10
Triathlon	15,664	PFC	1,221	Profix	906	Triathlon	7
AGC	8,575	Duracon	310	Legion	363	Duracon	5
Vanguard	8,297	Profix	235	AGC	194	Profix	5

Table 9. Proportions of different fixation methods in the Nexgen subgroup during 2000–2016

	Norway	Sweden	Finland	Denmark	Total (%)
Cemented	2,669	15,839	6,471	2,969	25,279 (85)
Uncemented	187	751	698	676	2,312 (8)
Hybrid	177	34	5	1,413	1,629 (5)
Inverse hybrid	1	123	341	32	497 (2)

Table 8. Additional Cox regression covariates with time-dependent coefficients (in different age groups and the Nexgen subgroup)

Factor	Follow-up (years)	Hazard ratio (95% CI)
**All patients aged < 65 years**		
Female sex	< 1	0.73 (0.66–0.82)
	1–3	1.15 (1.06–1.26)
	3–6	1.35 (1.19–1.52)
	> 6	0.98 (0.86–1.11)
Age per year	< 1	0.96 (0.95–0.97)
	1–3	0.95 (0.94–0.96)
	3–6	0.95 (0.94–0.96)
	> 6	0.95 (0.94–0.96)
Nation		
Norway	< 1	1.51 (1.27–1.79)
	1–3	1.64 (1.44–1.87)
	3–6	1.67 (1.39–2.00)
	> 6	0.98 (0.76–1.25)
Denmark	< 1	1.36 (1.16–1.59)
	1–3	1.53 (1.36–1.73)
	3–6	1.08 (0.90–1.29)
	> 6	1.52 (1.27–1.81)
Finland	< 1	1.32 (1.16–1.51)
	1–3	1.18 (1.06–1.31)
	3–6	1.05 (0.91–1.22)
	> 6	1.35 (1.17–1.56)
Sweden		1.0 reference
**Patients aged 55–65 years**		
Female sex	< 1.5	0.81 (0.74–0.90)
	1.5–3	1.24 (1.09–1.41)
	3–6	1.33 (1.14–1.54)
	> 6	0.99 (0.85–1.15)
Age		0.96 (0.95–0.97)
Nation		
Denmark		1.40 (1.28–1.53)
Finland		1.23 (1.14–1.33)
Norway		1.50 (1.36–1.66)
Sweden		1.0 reference
**Patients aged < 55 years**		
Female sex		1.07 (0.96–1.18)
Age		0.96 (0.95–0.97)
Nation		
Denmark	< 1	1.37 (1.01–1.85)
	1–3	1.86 (1.51–2.28)
	3–6	0.83 (0.60–1.16)
	> 6	1.22 (0.90–1.66)
Finland	< 1	1.55 (1.19–2.01)
	1–3	1.30 (1.06–1.58)
	3–6	1.01 (0.77–1.32)
	> 6	0.98 (0.75–1.28)
Norway	< 1	1.52 (1.08–2.13)
	1–3	1.91 (1.51–2.40)
	3–6	1.59 (1.15–2.20)
	> 6	0.81 (0.52–1.30)
Sweden	< 1	1.0 reference
	1–3	1.0 reference
	3–6	1.0 reference
	> 6	1.0 reference
**Patients aged < 65 years in the Nexgen subgroup**		
Female sex	< 1	0.61 (0.49–0.76)
	1–3	1.43 (1.18–1.72)
	3–6	1.34 (1.01–1.79)
	> 6	1.73 (1.15–2.61)
Age		0.95 (0.94–0.96)
Nation		
Denmark		1.84 (1.57–2.16)
Finland		1.39 (1.20–1.62)
Norway		1.62 (1.29–2.02)
Sweden		1.0 reference
